# Impact of salt stress, cell death, and autophagy on peroxisomes: quantitative and morphological analyses using small fluorescent probe N-BODIPY

**DOI:** 10.1038/srep39069

**Published:** 2017-02-01

**Authors:** Deirdre Fahy, Marwa N. M. E. Sanad, Kerstin Duscha, Madison Lyons, Fuquan Liu, Peter Bozhkov, Hans-Henning Kunz, Jianping Hu, H. Ekkehard Neuhaus, Patrick G. Steel, Andrei Smertenko

**Affiliations:** 1 Institute of Biological Chemistry, Washington State University, Pullman, WA-99164, USA; 2 Department of Genetics and Cytology, National Research Center, Giza, Egypt; 3 Plant Physiology, University of Kaiserslautern, Erwin Schrödinger Straße, Kaiserslautern D-67653, Germany; 4 Institute for Global Food Security, School of Biological Sciences, Queen’s University Belfast, 18-30 Malone Road, Belfast, BT9 5BN, UK; 5 Department of Chemistry and Biotechnology, Uppsala BioCenter, Swedish University of Agricultural Sciences and Linnean Center for Plant Biology, PO Box 7015, SE-75007 Uppsala, Sweden; 6 School of Biological Sciences, Washington State University, Pullman, WA-99164, USA; 7 MSU-DOE Plant Research Laboratory, Michigan State University, 612 Wilson Road, East Lansing, MI 48824, USA; 8 Department of Chemistry, Durham University, Durham, DH1 3LE, UK

## Abstract

Plant peroxisomes maintain a plethora of key life processes including fatty acid β-oxidation, photorespiration, synthesis of hormones, and homeostasis of reactive oxygen species (ROS). Abundance of peroxisomes in cells is dynamic; however mechanisms controlling peroxisome proliferation remain poorly understood because measuring peroxisome abundance is technically challenging. Counting peroxisomes in individual cells of complex organs by electron or fluorescence microscopy is expensive and time consuming. Here we present a simple technique for quantifying peroxisome abundance using the small probe Nitro-BODIPY, which *in vivo* fluoresces selectively inside peroxisomes. The physiological relevance of our technique was demonstrated using salinity as a known inducer of peroxisome proliferation. While significant peroxisome proliferation was observed in wild-type *Arabidopsis* leaves following 5-hour exposure to NaCl, no proliferation was detected in the salt-susceptible mutants *fry1-6, sos1-14,* and *sos1-15*. We also found that N-BODIPY detects aggregation of peroxisomes during final stages of programmed cell death and can be used as a marker of this stage. Furthermore, accumulation of peroxisomes in an autophagy-deficient *Arabidopsis* mutant *atg5* correlated with N-BODIPY labeling. In conclusion, the technique reported here enables quantification of peroxisomes in plant material at various physiological settings. Its potential applications encompass identification of genes controlling peroxisome homeostasis and capturing stress-tolerant genotypes.

Peroxisomes are ubiquitous eukaryotic organelles with an essential role in key cellular processes including fatty acid β-oxidation, metabolism of reactive oxygen species (ROS), and biosynthesis of the phytohormones auxin, jasmonates, and salicylates^1^,^2^,^3^. Furthermore, the peroxisomal lumen contains enzymes essential for specific steps of the glyoxylate cycle, and the photorespiratory pathway. Oxidation of glycolate to glyoxylate in peroxisomes produces the bulk of cellular ROS^4^ resulting in a high concentration of ROS in the peroxisomal lumen. Consequently, peroxisomal components are at risk of sustaining oxidative damage^5^. In particular, singlet oxygen (^1^O_2_) and hydroxyl radicals (OH^·^) cause peroxidation of polyunsaturated fatty acids as well as nitrosylation, carbonylation, and oxidation of amino acids. The combination of these modifications results in peroxisomal dysfunction through irreversible damage to integral membrane and lumen proteins, loss of enzyme activity as well as reduction of membrane fluidity and permeability^6^,^7^.

Peroxisomes are protected from oxidative damage through the action of antioxidants including ascorbate and glutathione (GSH), which neutralize ROS non-enzymatically, and by a plethora of ROS-scavenging enzymes including catalase, dehydro- and monodehydro-ascorbate reductase, glutathione peroxidase and glutathione reductase, ascorbate peroxidase, peroxiredoxins, and superoxide dismutase (SOD)^2^,^8^. The abundance of these enzymes makes peroxisomes a key organelle conferring cytoprotection against cellular ROS under normal growth conditions. Under stress conditions, peroxisomes become essential for survival. In particular, peroxisomal catalase has been implicated in removing the majority of hydrogen peroxide generated in response to light, salt, and ozone stresses factors^9^. Correspondingly, catalase activity increases in drought-stressed wheat^10^, pea^11^, alfalfa^12^, maize^13^ and rice^14^; whilst catalase deficiency leads to ROS accumulation and increased susceptibility to high-light stress in tobacco leaves^9^. Similarly, the activity of SOD and of ascorbate peroxidases increases significantly during drought stress^11^. In line with this, salt-resistant tomato species and drought-tolerant wheat varieties contain less H_2_O_2_ and exhibit higher activity of catalase, SOD, and ascorbate peroxidase^14^,^15^. Lastly, copper tolerance in pea correlates with higher activity of peroxisomal catalase and SOD^16^.

In addition to increasing the activity of the peroxisomal ROS-scavenging mechanisms, stress factors also promote peroxisome proliferation^17^,^18^,^19^,^20^,^21^,^22^. It is thought that proliferation is triggered by ROS through transcriptional up-regulation of genes encoding peroxisomal proteins^19^,^21^,^23^,^24^, including Peroxins 1 and 10 (PEX1 and PEX10), which facilitate peroxisome biogenesis^23^. Two interrelated modes of peroxisome proliferation have been proposed thus far: fission or *de novo* formation from the endoplasmic reticulum^1^.

Fission is the best understood mode of peroxisome proliferation. Two mechanisms of fission have been discovered to date: in the first mechanism, Peroxin11 (PEX11) promotes peroxisome elongation followed by division mediated by the Dynamin Related Proteins 3A and 5B (DRP3A; DRP5B), and FISSION1^25^,^26^; the second mechanism depends on PEROXISOME AND MITOCHONDRIAL DIVISION FACTOR1 (PMD1)^27^. Despite significant progress in understanding the molecular players of the fission process, the upstream regulatory components that link ROS signaling with transcriptional activation of fission-related genes remain poorly understood. Plant genomes lack apparent homologs to the animal Peroxisome Proliferation-Activated Receptors (PPARs^28^) or the yeast Pip2p/Oaf1p and Adr1p proteins^29^,^30^,^31^, which activate the fission machinery in response to developmental and environmental cues. Therefore, plants have likely evolved unique mechanisms for regulating peroxisome abundance in cells. For example, control of PEX11-dependent fission in response to far-red light relies on a plant-specific receptor, Phytochrome A, which acts upstream of the highly evolutionary conserved transcription factor HYH (Elongated Hypocotyl 5 Homologue)^32^. Understanding the biology of peroxisomes in developmental and stress contexts requires a more thorough knowledge of molecular mechanisms controlling peroxisome proliferation.

Identification of the missing components of the peroxisome proliferation regulatory network would benefit from a forward genetic approach. However, progress in this direction is hindered by the lack of techniques for quantification of peroxisome abundance in plant organs and tissues. Although mutants with altered peroxisome morphology have been successfully identified using fluorescent protein-based markers^33^,^34^, genetic screening for mutants with altered peroxisome abundance or proliferation has not been feasible thus far. Identification of such mutants would advance knowledge on plant peroxisome biology as well as plant stress adaptation. Detection of peroxisomes is technically challenging and mainly achieved by electron microscopy (e.g. Usuda *et al*.^35^), immunolabeling with antibodies against peroxisomal proteins^36^, and live-cell imaging with fluorescent proteins fused to peroxisome-targeting signals^37^. All these approaches are time- and resource-consuming, and hardly applicable for high-throughput screening. In our previous work, we have shown that a small fluorescent probe, Nitro-BODIPY (N-BODIPY), specifically labels plant peroxisomes *in vivo* and can be used to image peroxisomes in cells using fluorescence microscopy^38^. Based on this discovery, we have developed a technique for quantifying peroxisomes in cell extracts using spectrofluorimetry. Here we demonstrated the suitability of this approach for genetic screens by testing known and newly identified mutant alleles.

## Results

### Detection of peroxisomes by N-BODIPY

**Figure 1:**
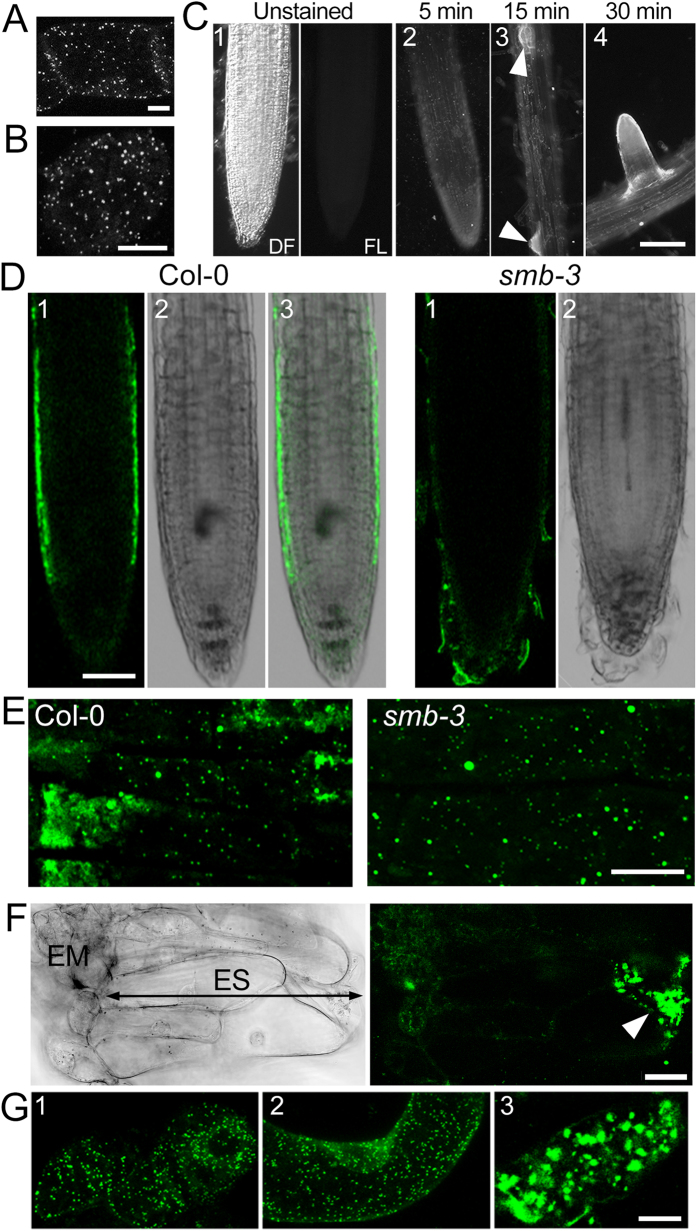
N-BODIPY staining of phylogenetically divergent plant systems. (**A**) *N. tabacum* BY-2 tissue culture cells. Scale bar 10 μm. (**B**) *A. thaliana* tissue culture cells. Scale bar 10 μm. (**C**) Time-course staining of *A. thaliana* roots. Arrowheads point to the secondary root primordia. (1–3), primary root; (4), secondary root. Images were acquired using identical acquisition parameters. Scale bar 250 μm. DF, dark field image; FL, fluorescence image. (**D**) Primary root of *A. thaliana* Columbia wild type (Col-0) and *smb-3* seedling after 2 h staining with 0.5 μM N-BODIPY. (1) fluorescence; (2) bright field; (3) merge. All images are 0.8 μm single optical sections. Scale bar 50 μm. (**E**) Peroxisomes in Col-0 and *smb-3* lateral root cap cells. Maximum projection of 5 optical sections each 0.8 μm thick; Scale bar 10 μm. (**F**) Bright field (left) and corresponding fluorescence image (right) of Norway spruce early embryos. Scale bar 50 μm. EM, Embryonal mass, ES, Embryo suspensor. Arrowhead points to cell at the late-stage of PCD on the distal end of the ES. (**G**) N-BODIPY staining of the embryonal mass cell (1), proximal suspensor cell, adjacent to the embryonal mass (2), distal suspensor cell (3). Scale bar 20 μm.

N-BODIPY was shown to label peroxisomes with high specificity in tobacco leaf and tissue culture cells as well as in *Arabidopsis* protoplasts^38^. Here, in addition to BY-2 cells (Fig. 1A) N-BODIPY was used to stain peroxisomes in *Arabidopsis* tissue culture cells (Fig. 1B) and roots (Fig. 1C and D). The fluorescence signal in root epidermis cells could be observed within only 5 min of incubation in N-BODIPY solution (Fig. 1C). The staining intensity increased further through the next 30 min of incubation. Confocal scanning laser microscopy detected the strongest signal in the lateral root cap cells (Fig. 1D). Whereas the lifespan of the epidermis cells equals the lifespan of the entire plant, root cap cells undergo programmed cell death (PCD) and peel off from the root tip^39^. ROS play an essential role in executing PCD and conceivably, more peroxisomes form during this stage. To test whether stronger staining, indicative of peroxisome proliferation, in the lateral root cup correlates with PCD, we used the SOMBRERO allele *smb-3* which exhibits defective PCD in the lateral root cap^39^. In agreement with our hypothesis, the staining of the lateral root cap was reduced in *smb-3* background compared to wild type (Fig. 1D). Detailed examination demonstrated three types of peroxisomes in the Col-0 background: normal sized, highly enlarged, and aggregated (Fig. 1E). While normal and enlarged peroxisomes were also observed in *smb-3* lateral root cap cells, no evidence for peroxisomal aggregation could be detected (Fig. 1E).

To examine the relevance of these aggregate peroxisomes in PCD, we took advantage of the developmental PCD during Norway spruce (*Picea abies*) embryogenesis. Typically, the embryo consists of two parts: the proliferating embryonal mass which eventually gives rise to the mature embryo and the terminally-differentiated embryonal suspensor which undergoes PCD. The suspensor is eliminated in the later stages of embryogenesis^40^. The proximal cells of the suspensor commit to PCD and undergo metabolic alterations that result in vacuolization of the cytoplasm and anisotropic cell expansion. The final stages of PCD, including vacuolar rupture and clearance of the cytoplasm content by lytic enzymes, take place in the distal cells. N-BODIPY stained the embryonal mass cells and the embryo suspensor (Fig. 1F). However, the strongest staining was detected in the distal cells that undergo the final stages of PCD (Fig. 1G). Taking into account the strong correlation between N-BODIPY staining of peroxisomal aggregates in cells undergoing death in two phylogenetically distant models, we conclude that N-BODIPY can be used as a PCD marker.

**Figure 2:**
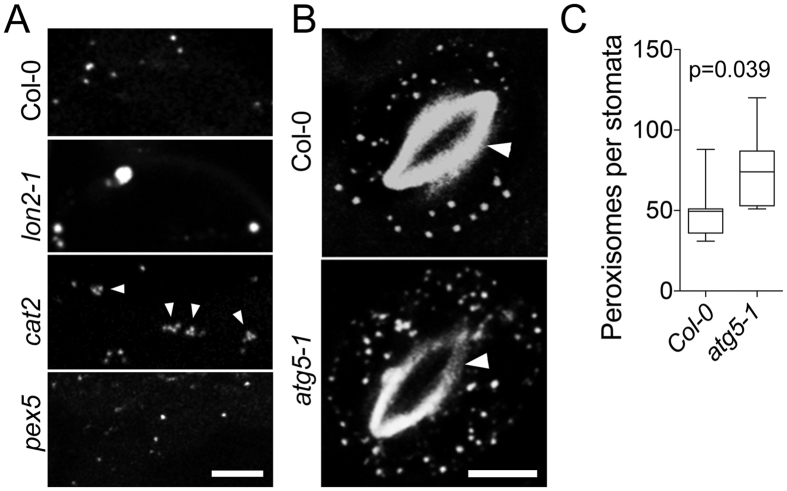
Staining of peroxisomes in mutants with known abnormalities of peroxisomal morphology or abundance. (**A**) N-BODIPY staining of mesophyll cells of Col-0, and mutant alleles of LON protease *lon2-1*, catalase *cat2*, and peroxisomal import receptor PEROXIN5 *pex5*. Arrowheads denote peroxisome aggregation. Scale bar 5 μm. (**B**) N-BODIPY staining of stomata cells in Col-0 and autophagy mutant *atg5-1*. Arrowheads indicate autofluorescence of the stomata cell walls. Scale bar 5 μm. (**C**) Number of peroxisomes per stomata in Col-0 and *atg5-1* background. Error bars show mean values of 8 biological repeats (n = 8) ± SD. p value was calculated by unpaired t-test with Welch’s correction.

Next we examined the ability of N-BODIPY to stain mutants with abnormal peroxisome biogenesis. A knockout of protease LON2 induces formation of enlarged peroxisomes in photosynthetic tissues^41^ and staining of mesophyll cells in *lon2* with N-BODIPY revealed enlarged peroxisomes (Fig. 2A). Second, a lack of catalase leads to peroxisomal aggregation^42^ and N-BODIPY also stained these aggregates in *cat2* mutants (Fig. 2A). Finally, peroxisomes are known to degrade *via* pexophagy, a type of selective autophagy^41^,^42^ and consequently, inhibition of autophagy in *atg5-1* mutants triggers accumulation of peroxisomes in cells^43^. In agreement with these observations, quantification of peroxisomes following N-BODIPY staining demonstrated ca. 50% increase in the number of peroxisomes per stomata (Fig. 2B,C) in *atg5-1* mutant plants relatively to Col-0 control. Therefore, N-BODIPY can be used for quantitative and qualitative analysis of changes in peroxisome size, shape, and localization.

### Quantification of peroxisomes in total cell protein extracts

**Figure 3:**
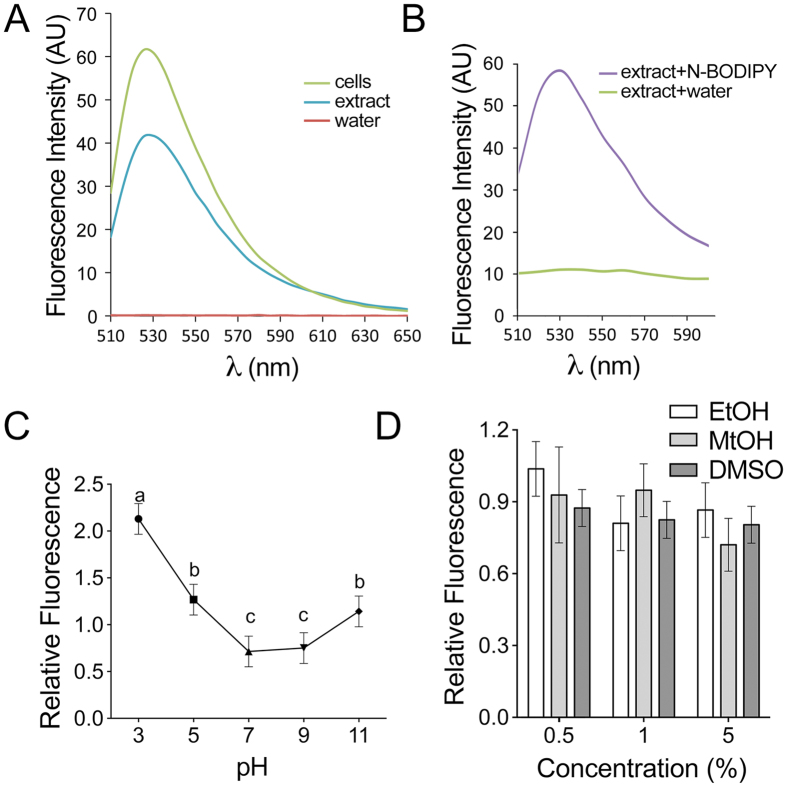
N-BODIPY fluorescence is activated by total protein extracts. (**A**) Representative chart showing emission spectra of 5 μM N-BODIPY incubated with: i. BY-2 cells (green); ii. total BY-2 protein extracts in water (blue); iii. water (red) at the excitation wavelength 490 nm. (**B**) Representative chart showing emission spectra of total protein extract from *Arabidopsis* tissue culture cells incubated with 5 μM N-BODIPY (violet) or with water (green) at 490 nm excitation. (**C,D**) Effect of pH (**C**) or solvents (**D**; Ethanol, white bars; Methanol, light-gray bars; DMSO, dark-gray bars) on N-BODIPY fluorescence in protein extracts. Error bars show mean values of three biological and three technical repeats ± SD. The difference between mean values denoted by the same letter is insignificant (P > 0.05, one-way ANOVA test). No significant differences between samples was found in (**D**). Values were normalized by the fluorescence of the protein extract in water.

Despite the potential to label peroxisomes in divergent model plant systems, application of N-BODIPY for labeling peroxisome abundance in plant material may be limited by access to fluorescence microscopes. Furthermore, counting peroxisomes by live-cell imaging is complicated by the rapid movement of peroxisomes. To overcome these limitations, we developed an assay to measure peroxisome proliferation in total cell extracts using spectrofluorimetry. Excitation of intact tobacco BY-2 suspension cells treated with N-BODIPY at 488 nm produced an emission spectrum with fluorescence maximum at 530 nm (Fig. 3A). Addition of N-BODIPY to total protein extract from BY-2 cells gave an identical emission spectrum. The fluorescence signal was below detectable level when water was used instead of the protein extract. The same fluorescence spectrum was recorded from total protein extract from *Arabidopsis* tissue culture cells (Fig. 3B) whereas omission of N-BODIPY resulted in a low background fluorescence signal (Fig. 3B). These data demonstrate that fluorescence of N-BODIPY is specifically activated not only in peroxisomes of living cells but also in total protein extracts.

Identifying sources of non-specific signal is critical during the development of fluorescence-based assays. Therefore, we examined the effect of protein extraction buffer composition on the intensity of N-BODIPY fluorescence in extracts. Whereas the fluorescence signal of the treated extract was increased by higher and lower pH (Fig. 3C), changing pH of a N-BODIPY solution in water without protein extract was insufficient to induce fluorescence. Based on these observations, we concluded that effective pH buffering in the assay is important to avoid artificial variability of N-BODIPY fluorescence. The ionic strength of the buffer did not have a significant effect on fluorescence for either K^+^ or Na^+^ (Supplemental Figure 1A). Finally, in agreement with our previous findings^38^, several commonly used organic solvents such as methanol, ethanol, and DMSO also had no significant effect on the fluorescence intensity (Fig. 3D). Hence, experimental compounds which would be dissolved in these solvents can be used for treating biological material prior to staining with N-BODIPY. Overall, the ability of protein extract to excite N-BODIPY fluorescence makes this approach amenable for high throughput applications in multiwell plate format.

### N-BODIPY binds to a protein target

**Figure 4:**
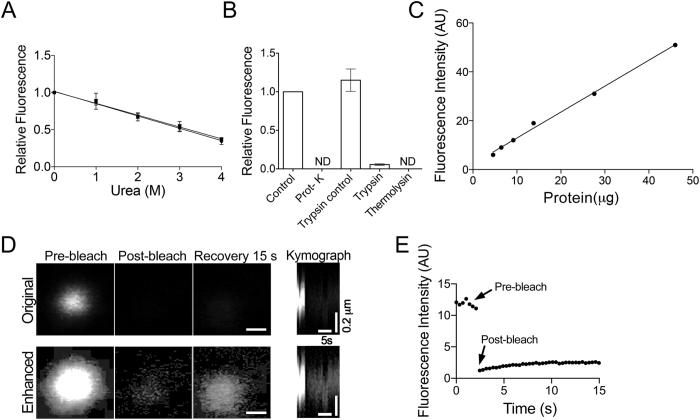
N-BODIPY binds stably to a protein target in peroxisomes. (**A**) Effect of protein denaturation with urea on N-BODIPY fluorescence in total protein extracts from BY-2 (squares) or *Arabidopsis* (circles) tissue culture cells. (**B**) Incubation with proteases abolishes N-BODIPY fluorescence. ND, not detected. The fluorescence signal was normalized by the control value. (**C**) Fluorescence signal is proportional to the protein content in the assay mixture. (**D**) Selected time frames and kymograph showing fluorescence signal in an individual peroxisome following photobleaching. Lower panel shows identical digitally enhanced images. Scale bar, 0.2 μm. (**E**) Quantification of fluorescence signal recovery of peroxisome shown in (**D**). Error bars show mean values ± SEM (N = 9).

The specific activation of N-BODIPY in peroxisomes suggests a requirement for a specific peroxisomal protein. In agreement with this hypothesis, denaturation of proteins in total extracts from BY-2 cells and *Arabidopsis* with 4M urea resulted in significant loss (ca. 50%) of the fluorescence intensity (Fig. 4A). Removal of urea from the samples by dialysis restored the fluorescence indicating that N-BODIPY target can reestablish its binding determinants during renaturation (Supplemental Figure 1B). Decreased fluorescence in Figure 4A is unlikely caused by urea quenching the fluorescence, because N-BODIPY lacks any detectable fluorescence in the absence of the extract (Fig. 3A). More likely, urea denatured the N-BODIPY target protein. If protein denaturation was the true reason for the fluorescence loss, then a elimination of the target by cleavage with proteases would have a similar effect. Indeed, treatment of the protein extract with proteases including Proteinase K (Prot-K), Trypsin, or Thermolysin resulted in complete or substantial reduction of the signal (Fig. 4B). Significantly, omitting the 37 **°**C incubation for Trypsin (Trypsin control) results in no loss of fluorescence intensity when compared to a ‘no Trypsin’ control. These experiments provide compelling evidence that N-BODIPY binds to a protein target.

As peroxisomes lack DNA, all their proteins must be imported into the lumen. Since N-BODIPY fluorescence in activated only in peroxisomes, we the examined effect of perturbed protein import on the labeling pattern. One of the best understood pathways of peroxisomal protein import depends on the receptor protein PEX5, which recognizes peroxisome targeting signal PTS1 (e.g. peptide SKL) and facilitates protein import into peroxisomes^1^. N-BODIPY staining of peroxisomes in *pex5* background allele (Fig. 2A) suggests that import of the N-BODIPY target is redundant with PTS1-dependent pathway.

Potentially, two mechanisms could exist for the activation of N-BODIPY fluorescence: stable association with the target or an enzymatic conversion of N-BODIPY from “dark” to fluorescent state. The latter case would complicate utilization of N-BODIPY for measuring peroxisome abundance as the signal would ultimately depend on this enzymatic activity and the reaction rate instead of target concentration. In our earlier work, analysis of the N-BODIPY in protein extracts failed to identify any metabolites suggesting that this scenario is unlikely^38^. We reason that to be a suitable probe for quantification, fluorescence of N-BODIPY must exhibit a linear correlation with the amount of peroxisomal protein and form a stable complex with its target. Fluorescence intensity of N-BODIPY was found to be proportional to the amount of total protein added to the assay mixture (Fig. 4C). To check the mode of N-BODIPY fluorescence activation, we used fluorescence recovery after photobleaching (FRAP) in BY-2 cells. Recovery of the signal would be consistent with an enzymatic conversion model, while no recovery would favor the stable binding model.

In agreement with the stable binding model, the bulk of N-BODIPY signal failed to recover (Fig. 4D). Digital enhancement of the images showed only minor recovery (Fig. 4D) estimated at ca. 10% of the initial signal (Fig. 4E) which likely reflects import of new N-BODIPY target into the peroxisome. To verify that lack of the recovery is not due to peroxisome movement, we digitally enhanced the gain to detect the remaining fluorescence and confirmed that peroxisomes remain stationary (Fig. 4E, Enhanced row). In conclusion, N-BODIPY appears to form a stable complex with its protein target. The fluorescence intensity correlates linearly with the concentration of this target.

### Evolutionary conservation of N-BODIPY target

**Figure 5:**
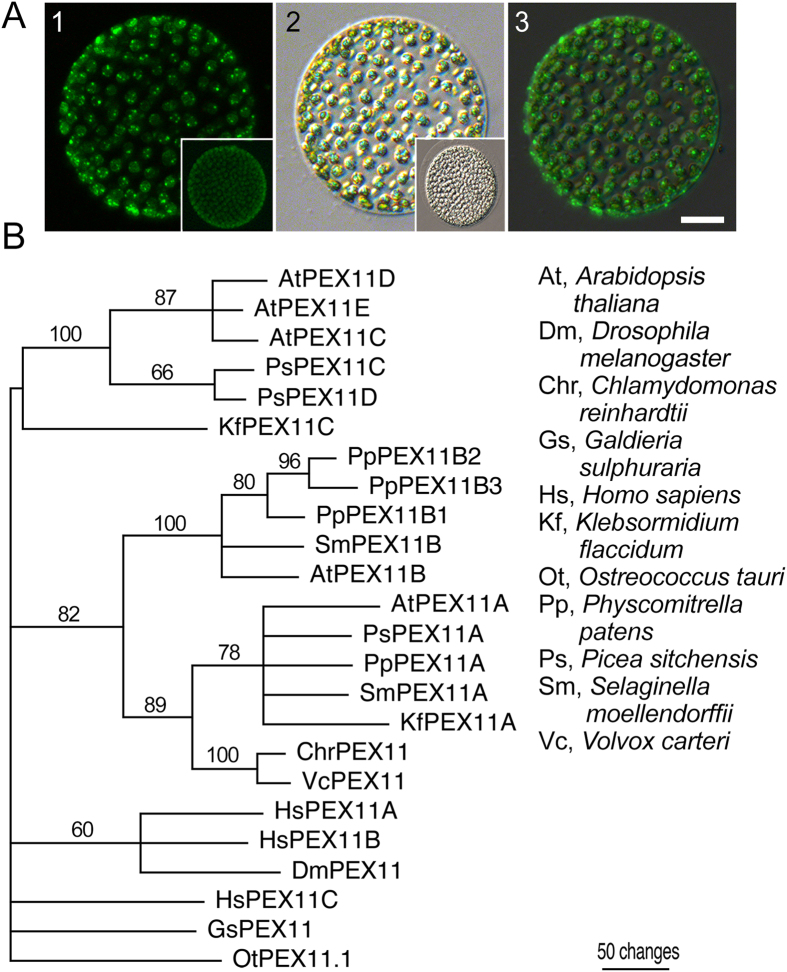
N-BODIPY staining of *Volvox sp*. (**A**) Fluorescence (1), bright field (2), and merged (3) images of *Volvox sp*. Scale bar 50 μm. Insets show unstained coenobium imaged at the same gain and exposure settings as the main images. (**B**) Phylodendrogram of a peroxisome biogenesis protein PEX11 from divergent lineages. A PEX11 homologue from *Ostreococcus tauri* was used as an outgroup. Accession numbers and alignment are provided in Supplemental Table 1 and Supplemental Dataset 1.

Staining of peroxisomes in several angiosperms and in the gymnosperm, Norway spruce, suggests that N-BODIPY may have wide application in plant research. However, our previous work demonstrated that N-BODIPY-positive compartments did not co-localize with peroxisomes in human cells^38^; therefore the target of N-BODIPY may be plant specific. To examine the extent of evolutionary conservation of the N-BODIPY target, we applied the probe to *Volvox sp.,* a representative of the Chlorophyta algae from the plant lineage that is distinct from land plants^44^. The staining pattern observed in the individual cells of the coenobium (Fig. 5A) was very similar to that of angiosperms and gymnosperms. To obtain a measure of the relatedness of peroxisomes in diverse plant species and non-plant outgroups, we conducted a phylogenetic analysis of a highly evolutionary conserved protein Peroxin 11 (PEX11) which localizes to peroxisomes and plays an essential role in peroxisome biogenesis^1^,^32^,^45^.

Representatives from several major phyla were included in this analysis. In addition to PEX11 homologue from *Volvox carteri*, we chose *Chlamydomonas reinhardtii* as another Chlorophycea (Green algae), and *Ostreococcus tauri* from Mamiellales order, a more distant Chlorophyta species. Major plant lineages were represented by the Charophyte *Klebsormidium flaccidum*, Bryophyte (Moss) *Physcomitrella patens,* Lycopodiophyta *Selaginella moellendorffii,* a gymnosperm *Picea sitchensis* and an angiosperm *A. thaliana.* As a representative species of distant to Chlorophyta algae, we used PEX11 from Rhodophyta (Red algae) *Galdieria sulphuraria.* Vertebrates were represented by *Homo sapiens* and invertebrates by *Drosophila melanogaster.*

The majority of PEX11 genes from Streptophyta (all Embryophytes and *K. flaccidum)* species formed three distinct clades on the phylodendrogram (Fig. 3B). One clade included *A. thaliana* PEX11 A, a second PEX11B, and a third PEX11C-E. We named genes in each clade after the corresponding *A. thaliana* homologues. The morphology of the phylodendrogram suggests that duplication of PEX11A resulted in the PEX11C clade and another duplication event of PEX11A resulted in PEX11B. Genomes of all analyzed members of Chlorophyta, *V. carteri, C. reinhardtii* and *O. tauri* encode only one PEX11. However, while PEX11 of *V. carteri* and *C. reinhardtii* sequences grouped together with PEX11A, the *O. tauri* PEX11 formed an independent clade when either *G. sulphuraria* or *D. melanogaster* sequences were used as an outgroup (Supplemental Figure 1). Sequences from other phyla, in particular human homologues, also formed independent clades in all three different outgroup experiments (Fig. 5B, Supplemental Figure 1), pointing out fundamental differences between plant and animal peroxisomes. The presence of a PEX11A homologue in *V. carteri* genome (BLAST score with *A. thaliana* PEX11A 179) suggests similarity between peroxisomes in algae and land plants. Conceivably, the target of BODIPY could be a protein which, like PEX11, has diverged in plant and animal lineages but is well conserved within plants or, alternatively, a plant-specific protein.

### Fluorescence intensity of N-BODIPY reflects peroxisome abundance

The correlation between peroxisome abundance and N-BODIPY in *atg5-1* and the linear relationship of fluorescence intensity of N-BODIPY with the amount of protein in the assay mixture makes N-BODIPY a useful probe for quantification of peroxisome abundance. The sensitivity of N-BODIPY to cellular peroxisome abundance was verified in experiments in which peroxisome proliferation was induced using clofibrate or hydrogen peroxide. Treatment with the lipoprotein lipase activator clofibrate is known to induce peroxisome proliferation^22^. We confirmed these observations using *A. thaliana* lines expressing CSY3-GFP, a peroxisomal marker. Treatment with 1 mM clofibrate for 5 hours resulted in 1.5 fold higher density of peroxisomes in the epidermis cells of root differentiation zone (Supplemental Figure 1C,D). Having shown that in our hands clofibrate treatment increases density of peroxisomes, we tested the ability of N-BODIPY to detect upregulation of peroxisome proliferation. We chose tobacco BY-2 suspension cells for this experiment because in liquid cultures all cells are homogenously exposed to the drug whereas in roots the epidermis cells would be exposed to a greater extent than underlying tissues.

**Figure 6:**
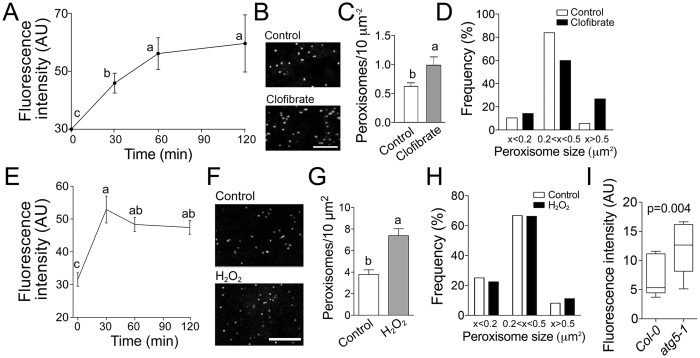
N-BODIPY fluorescence correlates with the number of peroxisomes in cells. (**A**) Time-course of N-BODIPY fluorescence in total extracts from BY-2 cells treated with 1 mM clofibrate. (**B**) N-BODIPY staining of cells treated with 1 mM clofibrate for 30 min. Scale bar 10 μm. (**C**) Density of peroxisomes in control and cells treated with clofibrate for 30 min (number of cells analyzed N=25). (**D**) Frequency of peroxisomal sizes. White bars, control; black bars, clofibrate (number of peroxisomes measured N = 150). (**E**) Time-course of N-BODIPY fluorescence in total extracts from BY-2 cells treated with 30 mM H_2_O_2_. (**F**) Staining of peroxisomes in cells treated with H_2_O_2_ for 30 min. Scale bar, 10 μm. (**G**) Density of peroxisomes in control and cells treated with H_2_O_2_ for 30 min (number of cells analyzed N = 29). (**H**) Frequency of peroxisomal sizes. White bars, control; black bars, H_2_O_2_ (number of peroxisomes measured N = 400). (**I**) N-BODIPY fluorescence in the total extracts from leaves of Col-0 and *atg5-1* plants (number of plants measured N = 5). Error bars show standard deviation. The difference between mean values denoted by the same letter is insignificant (P > 0.05, one-way ANOVA test in A and B, or t-test in C, G, and I).

The fluorescence signal increased rapidly after incubation with clofibrate, reaching a maximum at 60 min (Fig. 6A). Image analysis of cells stained with N-BODIPY after 30 min incubation with clofibrate showed ca. 30% increase in peroxisome density in the cytoplasm (Fig. 6B and C). In addition to increasing the overall number of peroxisomes, clofibrate treatment also caused an increase in the average peroxisomal size (measured as area of individual peroxisomal spots on 1 μm thick optical sections using Fiji image analysis software) from 0.28 μm^2^ to 0.34 μm^2^ (t-test p = 0.001, N = 150). The frequency of larger (area size over 0.5 μm^2^) and smaller (less than 0.2 μm^2^) peroxisomes (Fig. 6D) was higher in the clofibrate-treated cells. Similarly, treatment with hydrogen peroxide induced a significant increase of N-BODIPY fluorescence intensity after 30 min of treatment (Fig. 6E) and higher density of peroxisomes in the cytoplasm (Fig. 6F and G). However, in contrast to the clofibrate-treated cells, hydrogen peroxide treatment did not have any significant effect on the size of peroxisomes (Fig. 6H). In addition, we tested ability of N-BODIPY to detect changes in the peroxisome abundance due to the genetic defects. Autophagy mutant *atg5-1* is a useful model system to address this question because of higher number of peroxisomes in its cells (Fig. 2C). In agreement with the microscopy data, N-BODIPY fluorescence intensity in the total protein extracts from *atg5-1* leaves was higher, than in those from Col-0. Thus, N-BODIPY fluorescence in total extracts represents changes of peroxisome abundance in cells.

### N-BODIPY can be applied for genetic screens

As mentioned in the introduction, diverse abiotic stress factors can trigger peroxisome proliferation. Here we applied salt stress to test the suitability of N-BODIPY for monitoring plant response to the environment. A significant increase in the N-BODIPY signal was detected in leaf total protein extracts after 5 hours treatment of *Arabidopsis* plants with 0.3 M NaCl (Supplemental Figure 1E). Therefore, sensitivity of peroxisome proliferation to abiotic stresses can be used as a powerful phenotypic trait in screening for stress-susceptible or -tolerant mutants. To test this hypothesis, we measured peroxisome proliferation in response to salt stress in known and newly identified salt-sensitive alleles in *Arabidopsis*.

**Figure 7:**
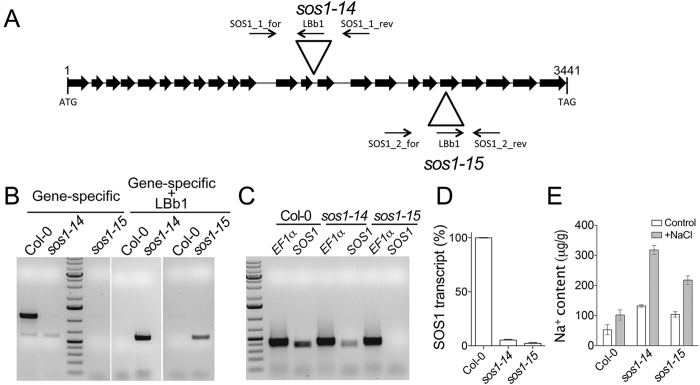
Characterization of *sos1* mutants. (**A**) Model of the *SOS1* gene and positions of T-DNA insertions. Arrows denote positions and directionality of corresponding genotyping primers. (**B**) Gel showing PCR fragments generated from genomic DNA of Col-0, *sos1-14,* and *sos1-15* with the combination of gene-specific or LBb1 and a gene-specific primer. (**C**) Na^+^ content in *sos1-14*, and *sos1-15* plants grown in control medium (Control) or medium supplemented with 2.5 mM NaCl for three days (+NaCl) was significantly higher than in Col-0 (P < 0.05, one-way ANOVA). Error bars show mean values of six biological repeats ± SEM.

We chose the *fry1-6* allele of inositol polyphosphate 1-phosphatase, an essential component of the stress signaling network, which is highly susceptible to salinity^46^. Furthermore, we also identified two new loss-of-function alleles of the proton/sodium antiporter SOS1^47^, *sos1-14* (Salk_114744) and *sos1-15* (Salk_149947). Positions of the tDNA insertion are shown in Fig. 7A. PCR-based genotyping demonstrated homozygous tDNA insertion in *sos1-14*, and *sos1-15* alleles (Fig. 7B). To check the effect of the *SOS1* knockout on intracellular sodium accumulation we grew plants on control liquid culture medium^48^ without additional sodium for five weeks and then supplemented the medium with 2.5 mM NaCl. Prior to salt addition both *sos1* alleles already had two fold higher Na^+^ content relative to the wild type plants (Fig. 7C). Incubation with 2.5 mM NaCl for three days resulted in an increase of Na^+^ in all plants; however, the Na^+^ content attained in *sos1* mutants was significantly higher than in Col-0 (Fig. 7C). These data indicate that both *sos1* alleles lack the ability to control Na^+^ homeostasis. A previously reported salt-susceptible knock-out tDNA allele of microtubule associated protein MAP65 *map65-1map65-2*^49^,^50^ was included in the analysis for comparison with other mutants.

**Figure 8:**
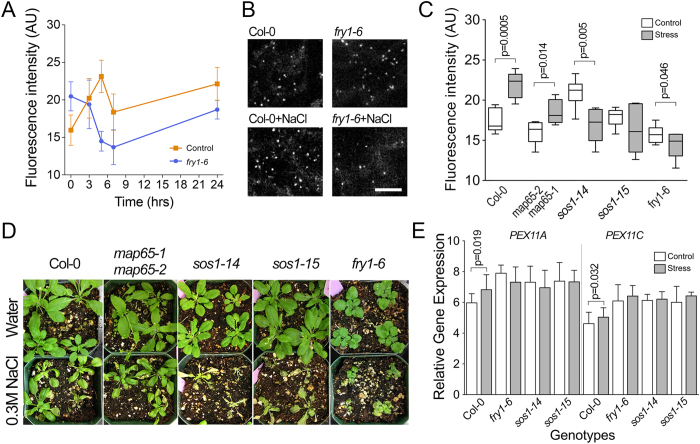
Peroxisome proliferation in salt-susceptible mutants. (**A**) Time-course of N-BODIPY fluorescence during salt-stress in Col-0 and *fry1-6* plants. Error bars show mean values of five biological repeats ± SD (n = 5). (**B**) N-BODIPY staining of leaf pavement cells in control and NaCl treated Col-0 and *fry1-6* seedlings. Leaves were imaged after 5 hrs of NaCl application. Scale bar 10 μm. (**C**) N-BODIPY fluorescence in total protein extracts from *Col-0, map65-1map65-2, sos1-14, sos1-15* and *fry1-6* leaves after treatment with water (white bars) or 0.3 M NaCl (gray bars) for 5 h. Error bars show mean values of five biological repeats ± SD (n = 5). p-values indicate the significance of the difference between treated and untreated samples in the unpaired t-test. (**D**) Representative images of control and NaCl treated plants taken 7 d after watering with water or 0.3 M NaCl respectively. (**E**) Quantification of PEX11 A and PEX11 C transcription level by qPCR in control and salt-stressed seedlings. Error bars show mean values of three biological and three technical repeats ± SD (n = 3).

Analysis of peroxisomes demonstrated significant differences between Col-0 and *fry1-6*: abundance increased in Col-0 peaking after 5 hours and progressively declined in *fry1-6* (Fig. 8A). In agreement with these data, staining of peroxisomes in leaf pavement cells of control and salt-treated (5 hours) plants also showed a lower density of peroxisomes in *fry1-6* than in Col-0 (Fig. 8B). No peroxisome proliferation was also detected in salt-susceptible mutants *sos1-14* and *sos1-15.* Furthermore, in *sos1-14* the peroxisomal signal was significantly lower than in untreated control. Unlike other mutants, *map65-1map65-2* showed similar proliferation of peroxisomes in response to NaCl as Col-0 Fig. 8C). Plant recovery was documented 7 days after watering with 0.3 M NaCl solution. While leaves of Col-0 and *map65-1map65-2* remained green, the leaves of all other mutants became chlorotic and developed lesions or signs of death (Fig. 8D).

Proliferation of peroxisomes was correlated with the transcription of two genes encoding known peroxisome biogenesis proteins PEX11A (AT1G47750) and PEX11C (AT1G01820)^51^. According to the public microarray data (AtGenExpress, http://jsp.weigelworld.org/expviz/expviz.jsp), transcription of both genes was upregulated by salt treatment. We also found upregulation of both transcripts after 5 hours of salt stress (Fig. 8E). However, no significant changes were detected in *fry1-6, sos1-14,* or *sos1-15* backgrounds. Interestingly, transcription of *PEX11A* and *PEX11C* was higher in these than in Col-0 even under non-stress conditions.

In conclusion, proliferation of peroxisomes is an important marker of salt-tolerance and measuring peroxisome proliferation using N-BODIPY can be successfully applied for discovery and analysis of genotypes with altered responses to salt stress.

## Discussion

### Application of N-BODIPY for quantification of peroxisomes

Small fluorescent live probes have become an essential tool in biological imaging. In comparison with traditional approaches based on the use of antibody or fluorescent proteins, detection of intracellular components using such probes offers several advantages. Firstly, small probes provide simplicity of use as they can penetrate the extracellular matrix and membranes detecting targets inside living cells within minutes of staining without the need for fixation or other preparatory steps. Secondly, the higher photo-stability and potentially higher density of labeling obtained allows for imaging at a lower intensity of excitation light resulting in reduced phototoxicity. Thirdly, compared to genetically encoded protein fluorescent markers, small molecular probes do not require genetic modifications and can be applied in a time- and tissue-specific manner. This enables any system to be analyzed in the laboratory or field settings. Over the last twenty years new probes have been developed for detection and quantification of a variety of essential cellular components such as metal ions, pH, ROS, ATP^52^,^53^.

BODIPY is currently one of the most popular fluorochromes for designing specific probes. This is based on its exceptional stability and quantum efficiency, and comprehensive knowledge about the effects of chemical modifications on the fluorescence characteristics^54^. Specific probes have been synthesized for detecting Cd^2+^, Zn^2+ ^55; Ca^2+ ^56; pH^57^; human adenosine-A1 and -A3^58^ and β-adrenoreceptors^59^. Previously, we have shown that N-BODIPY specifically labels peroxisomes in living plant cells^38^. However, accurate quantification of peroxisomes in plant organs is restricted by low permeability of N-BODIPY beyond the epidermis. Activation of N-BODIPY fluorescence by total protein extracts from plant material helps to overcome this constraint. Moreover, this approach can potentially be adapted for high-throughput analysis in multi-well plate format. The number of samples that could be analyzed using this technique is only limited by the availability of plant material.

Low fluorescence without protein extract implies that N-BODIPY becomes fluorescent upon binding to peroxisomal protein(s) rather than being simply accumulated in the peroxisomal lumen. Inhibition of the fluorescence by a high concentration of urea or by proteases indicates that a protein is responsible for the activation mechanism. Recovery of the signal after removal of urea by dialysis suggests that the protein target possesses a flexible secondary structure. Furthermore, the slow recovery of fluorescence after photobleaching is consistent with the high affinity of N-BODIPY to its binding site. The stability of the complex ensures consistent fluorescence intensity while measuring a large number of samples. Our data provide further insights into mechanisms of N-BODIPY fluorescence in plant cells and unveil its potential usage in detecting peroxisome abundance in plant tissues.

Considering the activity of N-BODIPY in phylogenetically divergent plant lineages, its protein target is likely to be involved in core functions of peroxisomes which had already evolved in the ancestral green flagellate ca. 700-1500Mya^44^ before the marine planktonic and freshwater/terrestrial lineage diverged. N-BODIPY did not co-localize with a marker of peroxisomes in human cells^38^ suggesting the target has diverged significantly during the evolution of the animal kingdom. Consistent with this hypothesis, the sequences of a conserved peroxisome biogenesis protein PEX11 has significantly diverged between animals and plants. Significant differences between core peroxisomal proteins are not surprising considering known roles of plant peroxisomes in photosynthesis, photorespiration, and synthesis of hormones. The target of N-BODIPY could be involved in a plant-specific peroxisomal process.

Peroxisomes contain enzymes that produce or decompose ROS during development and stress. Consequently, exogenously applied H_2_O_2_^24^ as well as stress-induced ROS^19^ trigger proliferation of peroxisomes demonstrating a positive feedback loop between ROS accumulation and activity of peroxisome proliferation machinery. Thus, peroxisome abundance serves as a proxy of ROS concentration. Measuring ROS content is time-consuming because each of the main ROS (singlet oxygen, superoxide radical, peroxide ion, hydrogen peroxide, and nitric oxide) requires a specific assay. Furthermore, the concentration of ROS in plant tissue undergoes rapid fluctuations due to their high reactivity. The abundance of peroxisomes in cells is a more consistent parameter than chemically unstable ROS. Therefore, combining peroxisome proliferation measurement with quantification techniques for determining concentration of specific ROS provides a reliable mean of assessing the overall status of ROS metabolism.

While quantification of gene transcription or protein expression offers crucial information about production of cellular molecules, it does not provide quantitative information on the abundance of specific cellular structures or organelles. Measuring content of structures or organelles in cells is essential for understanding cellular processes. For instance, quantification of DNA content in cells by flow-cytometry using DNA-specific fluorescent probes, Hoechst^60^ or DAPI^61^ has been instrumental for measuring endoreduplication and understanding its role in controlling cell size^62^. These studies demonstrated variability of ploidy from relatively modest in *Arabidopsis* (between 4N and 32N) to gigantic in *Phaseolus coccineus* (8,192C)^63^. Measuring peroxisomes in different mutants also demonstrated the potential of peroxisome abundance to undergo significant fluctuations. The most significant difference of ca. 60% reduction was observed between WT and *fry1-6* under salt stress. However, this difference is modest relative to the fluctuations of DNA content. Therefore, application of this technology to different experimental settings or to model systems would likely reveal a greater variability of peroxisome content.

### N-BODIPY detects late stages of programmed cell death

Small fluorescent probes can also be used to discriminate specific cell types. For example, probe KP-1 accumulates in pluripotent human cells because the expression of KP-1 efflux carriers is reduced in this cell type^64^. BODIPY-derived probe CDr3 specifically labels neural stem cells by interacting with the brain-specific fatty acid binding protein 7^65^. Some BODIPY-derivatives detect pathological conditions: a metalloproteinase-specific probe labels tumor, atherosclerosis, and inflammation cells^66^; β-amyloid probe labels plaques in diseased brain tissues^67^. In our experiments the intensity of N-BODIPY fluorescence was higher in cells undergoing final stages of PCD in external layers of the columella, the lateral root cap^39^, and distal embryo suspensor cells^40^. Currently, the late stage of PCD in plants can be recognized by detecting DNA fragmentation using terminal deoxynucleotidyl transferase dUTP nick end labeling (TUNEL) assay^68^. Labeling of peroxisomes provides a convenient cytoplasmic marker which complements TUNEL assay.

Similar staining with N-BODIPY in the embryonal masses and proximal suspensor cells demonstrates that peroxisome proliferation is not affected by the transition from proliferation to the early stages of PCD. However, the final stages of PCD are accompanied by pronounced proliferation of peroxisomes. As the proliferation of peroxisomes is controlled by cytoplasmic ROS, our findings indicate that ROS release also could occur in the course of developmental PCD. The majority of ROS in photosynthetic tissue is produced by coupled reactions in chloroplasts and peroxisomes^4^. However, as root cap and suspensor cells lack chloroplasts, ROS could be produced in mitochondria and then diffuse out through pores in mitochondrial membranes which become enlarged during cell death^69^,^70^. Previous studies have demonstrated that distal cells contain degraded nuclear DNA and ruptured lytic vacuoles^40^,^71^,^72^,^73^. This indicates that upregulation of peroxisome proliferation accompanies the degradation of the cytoplasm.

While the function of ROS in developmental PCD remains underappreciated, ROS are known to play a dual role in plant responses to abiotic stress and are one of the proximate causes of necrotic cell death^74^. At the early stages of stress response, ROS stimulate activation of tolerance mechanisms, while at the later stages, if tolerance failed to establish, they trigger cell death^75^. Salt stress (200 mM NaCl) in tobacco tissue culture cells causes induction of PCD through depolarization of the plasma membrane and reduction of intracellular concentration of K+^76^. The depolarization promotes the activity of NADPH-oxidase resulting in elevation of extracellular ROS within 1 hour of treatment^69^,^70^. It means that in contrast to the late ROS release during developmental PCD, stress is accompanied by rapid ROS increase during early stages. Diffusion of extracellular ROS into the cytoplasm ultimately leads to the loss of mitochondrial membrane potential (ΔΨ_m_), and opening of the permeability transition pores^77^,^78^. Leakage of mitochondrial ROS to the cytoplasm through the pores causes more oxidative damage to the cytoplasmic components. Therefore, one important aspect of plant adaptation to stress depends on preventing oxidative damage to the cellular components through upregulation of ROS scavenging mechanisms. For example, the salt-tolerant mutant of WITH NO LYSINE Kinase (*wnk8*) exhibited higher activity of peroxisomal catalase and peroxidase^78^.

### Proliferation of peroxisomes and salt-tolerance

Over-production of ROS in response to higher concentration of Na^+^ in the cytoplasm can be avoided by Na^+^ homeostasis mechanisms. In has been shown that extracellular Na^+^ diffuses into the cytoplasm through non-selective cation channels (NSCC). Consequently inhibition of NSCC with Zn^2+^ or Ln^3+^ reduces the salt-induced PCD and increases tolerance to salt stress^76^. The excess of cytoplasmic Na^+^ is removed through the Salt Overly Sensitive (SOS) signal transduction pathway^79^. The pathway becomes activated by a rapid intracellular Ca^2+^ increase occurring as a consequence of plasma membrane depolarization triggered by high concentrations of Na^+^. The intracellular Ca^2+^ signal is then perceived by a myristoylated calcium-binding protein SOS3. Calcium-bound SOS3 interacts with the protein kinase SOS2. The downstream effect of this interaction is activation of SOS2 kinase, phosphorylation of SOS3, and recruitment of SOS2 to the plasma membrane, where it activates a Na^+^/H^+^ antiporter SOS1. Activated SOS1 carrier proteins rapidly remove the excess Na^+^ from the cytosol.

Reduction of SOS1 activity diminishes the ability of cells to balance higher influx of Na^+^ resulting in lower salt tolerance. Both *sos1* knockout alleles used in this work exhibit significantly higher content of Na^+^ in the leaves when grown under normal conditions or under mild salinity. This observation is in line with the proposed function of SOS1 as a plasma membrane located Na^+^/H^+^ antiporter^47^. Normally SOS1 exports Na^+^ from root cells following nonspecific uptake. However, the lack of SOS1 activity in the knockout lines leads to increased root-to-shoot transfer of this toxic element^80^. In agreement with these data, both novel *sos1* alleles identified here exhibit salt susceptibility and also fail to increase peroxisome abundance.

Salt-susceptibility also correlated with reduction of peroxisome proliferation in *fry1-6* line. *FIREY1/SAL1* complements salt sensitivity of yeast^81^ and is required for tolerance to abiotic stresses in *Arabidopsis*^46^. It encodes a nucleotide phosphatase that *in vitro* has inositol polyphosphate 1-phosphatase and 3′,(2′),5′-bisphosphate nucleotide phosphatase activities^81^. The latter activity converts PAP (3′-polyadenosine 5′-phosphate) into AMP and phosphate (Pi)^81^,^82^. As PAP negatively regulates RNA silencing-suppressors XRN2, XRN3, and XRN4, its decomposition inhibits silencing activity^83^. Therefore, despite different mechanisms underlying salt-susceptibility in *fri1-6* and *sos1* alleles, all exhibited a lack of peroxisome proliferation under salt stress. This fact underlines the universal nature of peroxisome proliferation as a sensitive marker of early salt-susceptibility events.

Gene transcription analysis revealed correlation of peroxisome abundance with the transcription of *PEX11A* and *PEX11C* in Col-0. However, reduction of peroxisome abundance under salt stress in *sos1-14, sos1-15,* and *fry1-6* was accompanied by relatively constant *PEX11A* and *PEX11C* transcription level. Moreover, under normal growth conditions, transcription of both *PEX11* genes was higher in the mutants than in Col-0. These findings suggest that: (1) PEX11-dependent proliferation of peroxisomes requires activity of additional signaling pathways, which remains inactive in the mutant background and (2) salt stress could promote peroxisomal catabolism which causes reduction of peroxisome abundance in mutants with suppressed peroxisome proliferation.

The role of peroxisome proliferation in response to salinity as well as other abiotic stresses remains poorly understood. Higher activity of peroxisomal ROS-scavengers is known to correlate with tolerance to drought, salinity, and heavy metals^11^,^13^,^14^,^15^,^16^. Additionally, stresses such as salinity^21^, high light intensity^17^, ozone^18^,^19^, and metals^20^ induce proliferation of peroxisomes. However, artificial induction of peroxisome proliferation by over-expressing Peroxin 11 (PEX11) does not confer tolerance to higher salt concentrations^21^. More work is required to resolve whether peroxisome proliferation is a mere marker of stress or an integral part of the tolerance mechanism.

The contribution of peroxisomes to stress adaptation remains poorly understood because correlation between activity of peroxisomal enzymes and abundance of peroxisomes has not been systematically explored. The progress in addressing this important biological question is hindered by the technological gap in measuring proliferation of peroxisomes. The technique developed here is amenable to high-throughput detection of peroxisome proliferation in response to hydrogen peroxide, clofibrate, salt stress, and hyper-accumulation of peroxisomes due to the suppression of autophagy. Moreover, we found that N-BODIPY staining can be used as a marker for final stages of programmed cell death. As proliferation of peroxisomes is controlled by ROS production^19^,^24^, quantifying peroxisomes can potentially be used for monitoring status of ROS metabolism during development and stress situations. In addition to measuring peroxisome abundance under stress conditions or discriminating final stages of PCD, our technology can be applied in divergent plant lineages for predicting the outcome of the interaction between genotype and environment; for isolating stress-tolerant mutants; and for dissecting molecular mechanisms regulating proliferation of peroxisomes.

## Experimental Procedures

### Plant material, growth conditions, stress application and sodium quantification

*Arabidopsis* wild type Col-0 and mutant (Table 1) plants were grown in pots filled with 300 g of soil at 14/10 h light/dark cycle at 21 **°**C in the growth chamber. The salt stress was induced by watering each pot with 50 ml of 0.3 M NaCl solution in water. The treatment was carried out between 2 and 4 hours of the light period. Leaves (positions 5–9) were collected after 5 h. For root staining experiments, seeds were sterilized and germinated on half-strength MS salts, pH5.7, 0.7% plant agar (both from PhytoTechnology Laboratories).

*Arabidopsis* T87 cell line was maintained in NT-1 medium under continuous light at 24 **°**C and shaking at 130 rpm^84^. BY-2 cells were maintained in MS medium in the dark at 25 **°**C and shaking at 140 rpm^85^. Norway spruce somatic embryogenesis was induced by transferring one-week-old proliferating cell suspension to liquid half-strength LP medium devoid of auxin and cytokinin^40^.

For quantification of sodium accumulation *Arabidopsis* plants were grown in a hydroponic culture system as reported^48^ and cultivated in a growth chamber at 20 °C at light intensity 125 μmol photons m^−2^ sec^−1^ in a 10 h light/14 h dark regime. After 5 weeks of growth, the growth medium was exchanged for fresh medium supplemented with 2.5 mM NaCl for a further 72 h. In the control samples NaCl was omitted from the medium. Leaves were harvested, ground in liquid nitrogen and the tissue powder was then resuspended in double-distilled water and centrifuged at 20,000 g for 10 min at room temperature to remove cell debris. Ion content was measured in the supernatant using a 761-IC compact system (Metrohm)^86^.

For inducing peroxisome proliferation in BY-2 cells, 1 ml of one-week-old cell culture was subcultured to 250 ml flask containing 60 ml of fresh medium. On the fourth day, 10 ml of the culture was collected as time point 0 and hydrogen peroxide or clofibrate were added to a final concentration of 30 mM and 1 mM, respectively. At each time point, 10 ml of the cell culture were collected for measuring peroxisomes. The cells were immediately centrifuged, then the supernatant was aspirated and the pellet was frozen in liquid nitrogen.

### Genetic analysis and generation of mutants

The *Arabidopsis sos1* T-DNA insertion mutants Salk_114744 (*sos1-14*) and Salk_149947(*sos1-15*) were obtained from the Nottingham Arabidopsis Stock Centre (http://arabidopsis.info). For genotyping of *sos1* mutants, genomic DNA was isolated from four-week-old *Arabidopsis* plants and used for PCR. Corresponding primers are listed in Supplemental Table 2.

### Protein extraction and N-BODIPY fluorescence assays

Tissue culture cells were collected by centrifugation at 500 rpm for 1 min at room temperature. *Arabidopsis* leaves were collected from five-week-old plants. Plant material was ground in liquid nitrogen and ca. 100 mg of the ground powder was used for protein extraction. The total protein was extracted by adding ddH_2_O or extraction buffer containing 50 mM Tris-HCl, pH8.0, 0.5 M NaCl, 8 M urea. Urea was added to improve the extraction efficiency. The final concentration of urea in the assay was 0.4 M. The effect of this concentration on N-BODIPY fluorescence was found to be statistically insignificant. Cell debris was removed by centrifugation for 10 min at 14,000 rpm at room temperature. Then a 10 μL aliquot of the extract was supplemented with 190 μL of 2 μM solution of N-BODIPY (Nitro-4,4-Difluoro-4-bora-3a,4a-diaza-s-indacene) in 96-well plates and incubated for 10 min. For testing different buffer conditions, 0.1 ml of 2 μM N-BODIPY solution was mixed with 10 μL of the extract and 90 μL of corresponding reagents to reach the desired conditions. The N-BODIPY solution was freshly prepared in water from 10 mM stock in DMSO. The fluorescence intensity was measured at 490 nm excitation wavelength and 530 nm emission wavelength using spectrofluorimeter Synergy Neo B (Biotek Instrument, Inc). The background signal was determined by: 1) measuring the N-BODIPY signal with 10 μL of Extraction buffer, and 2) measuring 10 μL of the extract in 190 μL of water. Both background values were subtracted from the signal value. The fluorescence signal was normalized by the protein concentration in the extracts measured with the Bradford assay using a calibration curve constructed with solutions of known concentration of BSA (Bovine Serum Albumin). Fluorescence intensity was calculated in arbitrary units per 1 mg of protein in the extract. At least three biological repeats and three technical repeats were performed per each sample.

Three proteases were used in the experiments: Proteinase K (800 U/ml; New England Biolabs, Inc), Thermolysin, (55U/mg; SigmaAldrich, Co, LLC); and Trypsin (3000 U/ml; SigmaAldrich, Co. LLC). The reaction contained 1.2 U of Proteinase K, 2 U of Thermolysin, or 240U of Trypsin and 20 μl of total protein extract in water. After incubation at 37 **°**C for 1 h, the digestion mixtures were mixed with 100 μl of 5 μM N-BODIPY and fluorescence was measured at 490 nm excitation and 530 nm emission wavelengths. Proteinase K and Thermolysin were diluted in TE-buffer (10 mM Tris, pH 8.0, 1 mM EDTA), trypsin was diluted in 1 mM EDTA solution, pH 7.5 to final volume 80 μl. Then each tube was supplemented with 20 μl of total protein extract from *Arabidopsis* leaves in water and incubated at 37 **°**C for 1 hr. For the negative controls, incubation at 37 **°**C was omitted. In the positive controls, the corresponding buffers were used without proteases.

### Microscopy and image analysis

To stain peroxisomes in liquid cultures, N-BODIPY was added to a final concentration of 1 μM from 10 mM solution made in DMSO. The images were acquired after 15 min. Quantification of peroxisomes in images collected from living cells is complicated by the rapid and stochastic movement of peroxisomes. To overcome this problem, we used resonant scanning mode (12000 Hz) of the Leica SP8 confocal microscope, 512 × 512 image resolution, four averages, at image acquisition rate of 0.1s per frame. Peroxisome density and size were calculated on the maximum projection images composed of three 1 μm-thick optical sections using Particle Analysis tool of Fiji (http://fiji.sc/Fiji). The same image acquisition mode was used in FRAP (Fluorescence Recovery After Photobleaching) experiments except that only immobile peroxisomes were selected. 70% laser power for three consecutive frames was used during the bleaching step^87^. The signal intensity was measured using Fiji.

### Phylogenetic analysis

The PEX11 sequences (Supplemental Table 1) were aligned using the ClustalX software package^87^, and the phylodendrogram was constructed using the bootstrap resampling method of PAUP 4.0 software (Sinauer Associates). Bootstrap values were calculated from 5000 replicates and only groups with bootstrap scores 60 or above were retained in the phylodendrogram. *O. tauri* PEX11 was used as an outgroup. The alignment is included as Supplemental Data Set 1.

#### RNA extraction, cDNA synthesis, and qPCR

The RNA was extracted from leaves using the RNeasy plant mini kit (Qiagen Inc, CA, USA). The cDNA was synthesized using the Maxima H Minus First Strand cDNA Synthesis Kit (Thermo Fisher Scientific Inc.). The qPCR primers were designed using Primer-Blast and listed in Supplemental Table 2. Three biological replicates and three technical replicates for each sample were performed. The ΔC_T_mean was calculated using normalization with the reference gene AtEF1α.

## Additional Information

**How to cite this article**: Fahy, D. *et al*. Impact of salt stress, cell death, and autophagy on peroxisomes: quantitative and morphological analyses using small fluorescent probe N-BODIPY. *Sci. Rep.* **7**, 39069; doi: 10.1038/srep39069 (2017).

**Publisher's note:** Springer Nature remains neutral with regard to jurisdictional claims in published maps and institutional affiliations.

## Supplementary Material

Supplemental Tables and Figures

Supplemental Dataset File

## Figures and Tables

**Table 1:** Mutant lines used in this study.

Allele	Gene	Accession number	Polymorphism	Reference	Background	Donor
*pex5*	PEX5	AT5G56290	pex5_EMS	88	Col-0	Jainping Hu
*lon2-1*	LON2	AT5G47040	SALK_127495C	88	Col-0	ABRC
*cat2-1*	Catalase	AT4G35090	SALK_076998	89	Col-0	ABRC
*atg5-1*	Autophagy5	AT5G17290	CS39993	90	Col-0	Richard Vierstra
*smb-3*	SOMBRERO	AT1G79580	SALK_143526C	91	Col-0	ABRC
*sos1-14*	SOS1	AT2G01980	SALK_114744	N/A	Col-0	NASC
*sos1-15*	SOS1	AT2G01980	SALK_114744	N/A	Col-0	NASC
*fry1-6*	FIERY1	AT5G63980	SALK_020882	92	Col-0	Fuquan Liu
*map65-1map65-2*	MAP65-1, MAP65-2	AT5G55230, AT4G26760	SALK_006083, GK-849D05	50	Col-0	Michiko Sasabe
