# Corrigendum: Impact of salt stress, cell death, and autophagy on peroxisomes: quantitative and morphological analyses using small fluorescent probe N-BODIPY

**DOI:** 10.1038/srep46643

**Published:** 2017-05-03

**Authors:** Deirdre Fahy, Marwa N. M. E. Sanad, Kerstin Duscha, Madison Lyons, Fuquan Liu, Peter Bozhkov, Hans-Henning Kunz, Jianping Hu, H. Ekkehard Neuhaus, Patrick G. Steel, Andrei Smertenko

Scientific Reports 7: Article number: 39069; 10.1038/srep39069 published online: 02 01 2017; updated: 05 03 2017.

This Article contains errors. In Table 1, the polymorphism for *sos1-15* “SALK_114744” should read “SALK_149947”.

Additionally, the legend for Figure 7 is incorrect:

“(**A**) Model of the *SOS1* gene and positions of T-DNA insertions. Arrows denote positions and directionality of corresponding genotyping primers. (**B**) Gel showing PCR fragments generated from genomic DNA of Col-0, *sos1-14,* and *sos1-15* with the combination of gene-specific or LBb1 and a gene-specific primer. (**C**) Na^+^ content in *sos1-14*, and *sos1-15* plants grown in control medium (Control) or medium supplemented with 2.5 mM NaCl for three days (+NaCl) was significantly higher than in Col-0 (P < 0.05, one-way ANOVA). Error bars show mean values of six biological repeats ±SEM”.

should read:

“**A**, Model of the *SOS1* gene and positions of T-DNA insertions. Arrows denote positions and directionality of corresponding genotyping primers. **B**, Gels of PCR fragments generated from genomic DNA of Col-0, *sos1-14,* and *sos1-15* with the combination of gene-specific or LBb1 and a gene-specific primer. **C,D**, Gel of RT-PCR fragments generated from RNA of Col-0, *sos1-14,* and *sos1-15* with *SOS1* or *EF1α* primers (**C**); and normalized intensity of *SOS1* fragments (**D**). **E**, Na^+^ content in *sos1-14*, and *sos1-15* plants grown in control medium (Control) or medium supplemented with 2.5 mM NaCl for three days (+NaCl) was significantly higher than in Col-0 (P < 0.05, one-way ANOVA). Error bars show mean values of six biological repeats ±SEM”.

