# Supplementary material for: Impact of salt stress, cell death, and autophagy on peroxisomes: quantitative and morphological analyses using small fluorescent probe N-BODIPY
Source: Sci Rep. 2017 Feb 1;7:39069. doi: 10.1038/srep39069 (PMC5286434; doi:10.1038/srep39069)
Supplement: Supplemental Tables and Figures [file srep39069-s1.pdf]

SUPPLEMENTAL INFORMATION

Quantification Peroxisome Abundance in Plants Using Small Fluorescent Probe N-BODIPY

Deirdre Fahy, Marwa N.M.E. Sanad, Kerstin Duscha, Madison Lyons, Fuquan Liu, Peter Bozhkov, Henning Kuntz, , Jianping Hu, H. Ekkehard Neuhaus, Patrick G. Steel, Andrei Smertenko

Supplemental table 1. List of genes used for phylogenetic analysis.

Abbre-viation	Name	Group	Protein name	Accession number
At	*Arabidopsis thaliana*	Angiosperms	PEX11A	AT1G47750
			PEX11B	AT3G47430
			PEX11C	AT1G01820
			PEX11D	AT2G45740
			PEX11E	AT3G61070
Chr	*Chlamydomonas reinhardtii*	Chlorophyta (Green algae)	ChrPEX11	gi|159471834
Dm	*Drosophila melanogaster*	Invertebrates	PEX11	gi|19922346
Gs	*Galdieria sulphuraria*	Rhodophyta (Red algae)	GsPEX11	gi|545712326
Hs	*Homo sapiens*	Vertebrates	PEX11A	gi|4505717
Hs	*Homo sapiens*	Vertebrates	PEX11B	gi|296317239
Hs	*Homo sapiens*	Vertebrates	PEX11C	gi|18087833
Kf	*Klebsormidium flaccidum*	Charophyta	KfPEX11A	gi|971519848
			KfPEX11C	gi|971518404
Ot	*Ostreococcus tauri*	Chlorophyta	OtPEX11.1	gi|693496001
Pp	*Physcomitrella patens*	Bryophyta (Mosses)	PpPEX11A	gi|168027834
			PpPEX11B1	gi|168005036
			PpPEX11B2	gi|168041391
			PpPEX11B3	gi|168008697
Ps	*Picea sitchensis*	Gymnosperm	PsPEX11C	gi|116791870
			PsPEX11D	gi|116779031
Sm	*Selaginella moellendorffii*	Lycopodiophyta	SmPEX11A	gi|302808231
			SmPEX11B	gi|302780655
Vc	*Volvox carteri f. nagariensis*	Chlorophyta	VnPEX11	gi|302848496

Supplemental Table 2. List and sequences of primers.

Primer Name	Sequence
SOS1_1_for	TCATGGCGATTTCTTTTTCTG
SOS1_1_rev	TCCCAGTAAGTTGCTTGCA
SOS1_2_for	GCTACATTTCTGCTGCGTTTC
SOS1_2_rev	ATCAAGTCGCACAGATATGGC
LBb1	GCGTCGACCGCTTGCTGCAACT
ATPEX11A-F	TGCTTGGGCTGAATTGGTTG
ATPEX11A-R	CTCGATCGTCGAAGCAACAC
ATPEX11C-F	GCTTGGCAGGACTGGGATTT
ATPEX11C-R	GCTCCCCAACCTCAACCAAG
At EF1a.F	TGGTGACGCTGGTATGGTTA
At EF1a.R	TCCTTCTTGTCCACGCTCTT

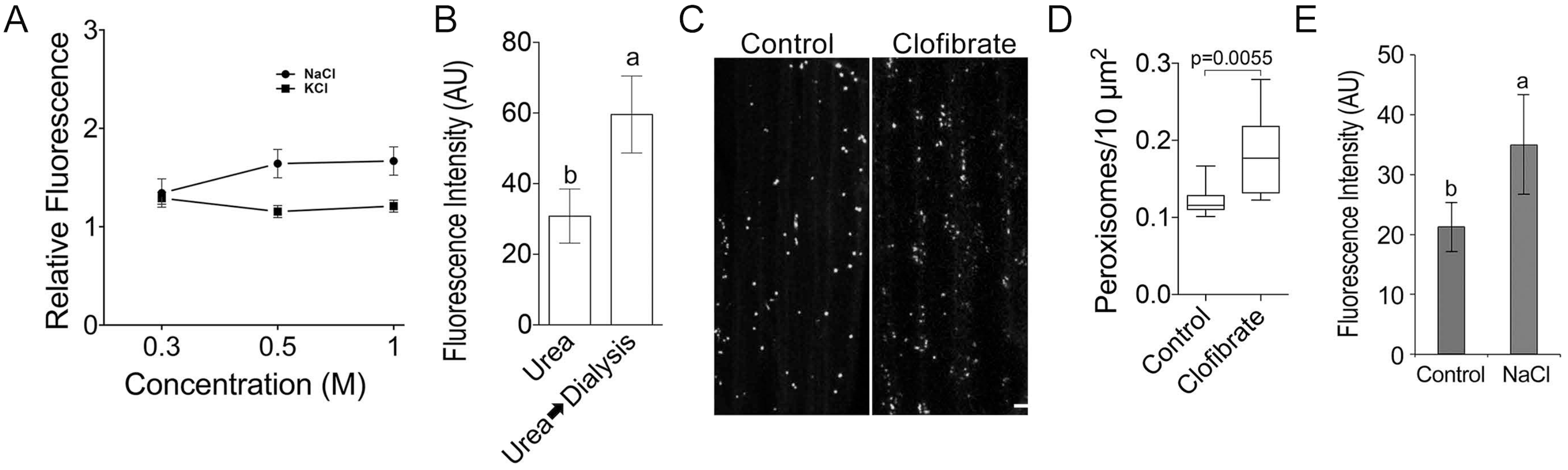

Supplemental Figure 1. Assays of N-BODIPY fluorescence and peroxisome proliferation.
A, Effect of NaCl (circles) or KCl (squares) on N-BODIPY fluorescence in protein extracts. Error bars show mean values of three biological and three technical repeats ±SD. The difference between the measurements was not statistically significant (one-way ANOVA; N=6). Values were normalized by fluorescence values of the protein extract in water.
B, Recovery of N-BODIPY fluorescence following denaturation by 4 M urea. The total protein extract was supplemented with 4 M urea (Urea) and then dialyzed against distilled water for 16 hrs (Urea Dialysis). Error bars show mean values of three technical repeats ±SD.
C, Effect of clofibrate on peroxisomes. Fluorescence microscopy images of root epidermis cells in the differentiation zone of *A. thaliana* seedlings expressing CSY3-GFP. Scale bar 10 mm.
D, Effect of clofibrate on the density of peroxisomes (calculated per 10 mm^2) in epidermis cells. Error bars show mean values of ten biological repeats ±SD; p-value indicate the significance of the difference between treated and untreated sample in the unpaired t-test with Welch's correction (n=20).
E, Fluorescence of N-BODIPY in total protein extract from *Arabidopsis* leaf 5 hours after irrigation with water or 0.3 M NaCl. Error bars show mean values of five biological repeats ±SD. The difference between mean values denoted by the distinct letter is statistically significant ($P<0.05$, t-test).

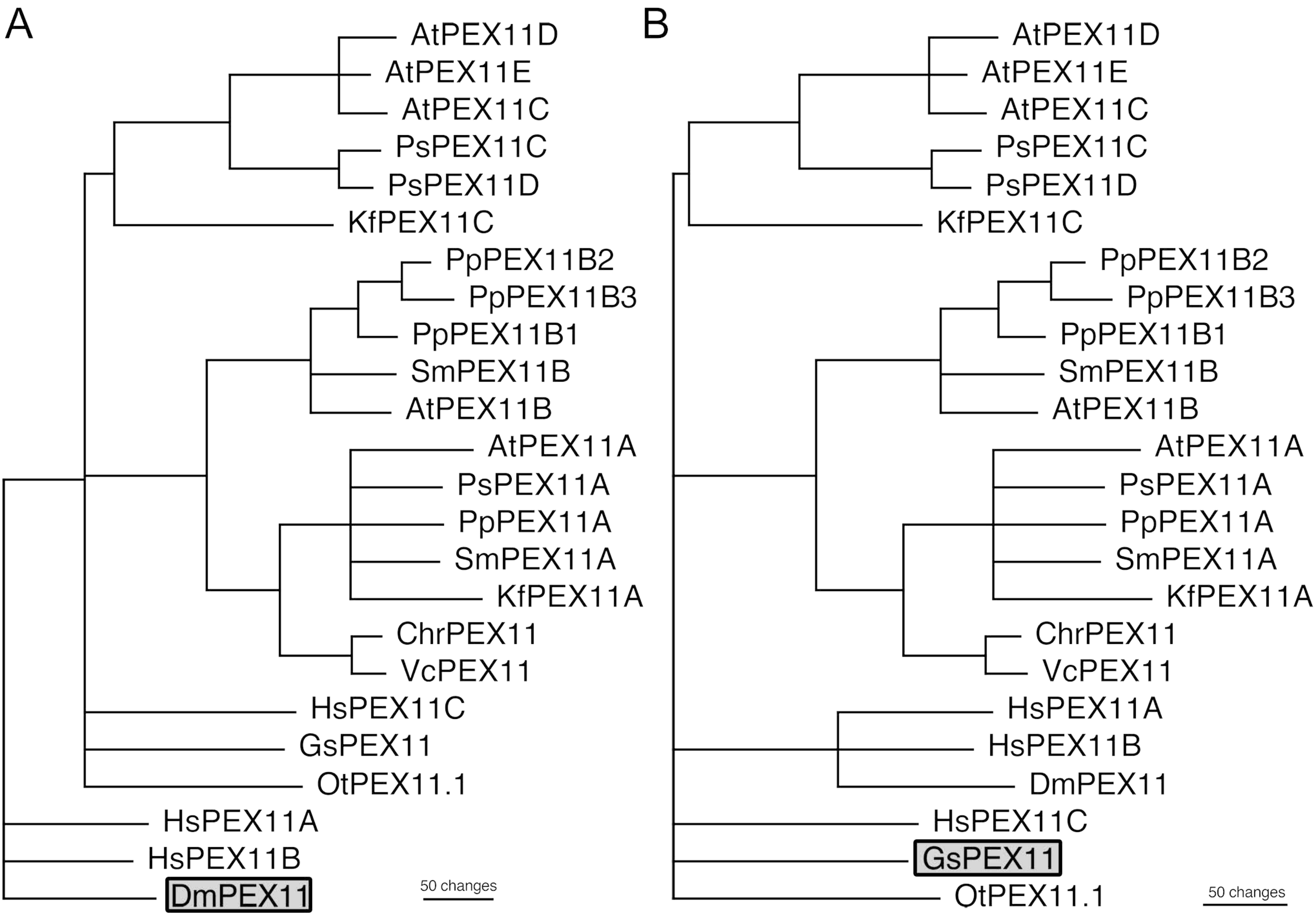

Supplemental Figure 2. Phylodendrograms of PEX11 genes.
A, *Drosophila melanogaster* PEX11 was used as an outgroup.
B, *Galdieria sulphuraria* PEX11 *was used as an outgroup.*
The outgroup sequences are highlighted.
